# Analysing the Influence of Speed and Jumping Performance Metrics on the Percentage Change of Direction Deficit in Adolescent Female Soccer Players

**DOI:** 10.3390/life14040466

**Published:** 2024-04-03

**Authors:** Alberto Roso-Moliner, Oliver Gonzalo-Skok, Víctor Emilio Villavicencio Álvarez, Santiago Calero-Morales, Elena Mainer-Pardos

**Affiliations:** 1 Health Sciences Faculty, Universidad San Jorge, Autov. A23 Km 299, Villanueva de Gállego, 50830 Zaragoza, Spain; aroso@usj.es (A.R.-M.); epardos@usj.es (E.M.-P.); 2 Department of Communication and Education, Universidad Loyola Andalucía, 41704 Seville, Spain; oligons@hotmail.com; 3 Department of Human and Social Sciences, Universidad de las Fuerzas Armadas-ESPE, Quito 171103, Ecuador

**Keywords:** sprint, football, women, multidirectional speed, team sport

## Abstract

Studies show that although female soccer players often have shorter change of direction (COD) deficits than males, indicating different biomechanical profiles, there is a lack of research on the impact of physical metrics on COD performance in females. The purpose of this work was to analyse whether performance metrics based on speed and jumping could explain the variation in %CODD in young female soccer players. Thirty-three highly trained adolescent female soccer players with an age of 16 ± 0.95 years, a body mass of 55.7 ± 7.22 kg, and a height of 160.4 ± 5.22 cm performed COD180 tests, 10 m and 30 m sprint tests, single-leg countermovement, and horizontal jumps. Acceleration in the first 10 m of a sprint was identified as a significant predictor of COD180 performance (R^2^ = 28%), (R^2^ = 50%), (*p* < 0.01), indicating that early sprint performance may largely determine an individual’s ability to change direction. However, no predictors were found for %CODD. Significant correlations were observed between COD180 performance and %CODD, acceleration, linear speed, and horizontal jump performance (*r* = −0.59 to 0.70; *p* < 0.05). The study suggests that specific physical performance metrics, particularly early acceleration, are crucial for enhancing COD skills in female soccer players, emphasizing the need for targeted training interventions.

## 1. Introduction

Women’s soccer has grown exponentially in recent decades, with an increase of just over 24% from 2019 to 2023. During this time, 16.6 million women played soccer, with 3.9 million federated female players (19,064 of these players are professionals), 55,622 women’s clubs, and 48,202 female coaches [1]. Interest has also increased at the social and institutional level, and, in 2019, FIFA (Fédération Internationale de Football Association) presented the “Women’s Football Strategy” [2]. Among the objectives of this strategy are to encourage the growth of the sport, to attract more female players, and to enable them to play for more years [2].

Soccer, as an intermittent sport, involves several brief, high-intensity actions that are repeated throughout a match, including shots on goal, sprints, jumps, and acceleration and deceleration. A significant part of these actions requires changes of direction (CODs), which are decisive in determining the outcome of a match and serve as reliable indicators of performance levels in the sport [3]. These CODs require players to accelerate, decelerate, and change direction as quickly as possible. Previous research highlights the importance of linear speed [4,5,6], lower limb power [3,7], reactive strength [8,9], motor control [8], or a combination of these components [10] in improving COD performance. At the same time, the nature of soccer demands unilateral movement skills and usually involves multidirectional activities such as jumping or cutting [11]. Actions such as unilateral and bilateral jumps, linear and curvilinear sprints, zig-zag runs, side steps, cross-cuts, and repeated back-and-forth movements are common [11]. In particular, direct sprinting often becomes a critical action during goal or assist phases among professional female players. In competitive scenarios, elite female soccer players are known to cover a total distance of 9–11 km, with 590–840 m at high intensities of 15.6–20 km/h and 198–379 metres via sprints at speeds above 20 km/h [12].

The change of direction deficit (CODD), introduced by Nimphius et al. [13], serves as a measure to analyse COD capacity by emphasising the specific timing of COD actions, offering a more accurate comparison between groups through a percentage-based approach [14]. This method suggests a closer mirroring of actual COD performance, revealing that female athletes, particularly female soccer players, often exhibit shorter CODD than men, indicating sex-specific biomechanical and performance profiles [15,16]. Kobal et al. [17] found that athletes with higher sprint speeds may have greater CODD, implying that high speed does not necessarily correspond to efficient COD capacity. Female soccer players, in particular, show greater CODD than those in other team sports, suggesting lower efficiency in directional change despite their sprinting abilities [18]. The relationship between CODD and jumping power has also been explored, showing that better jumping ability correlates with better CODD performance in female athletes [7,8,19], reinforcing the value of CODD in the assessment and training of female soccer players. These results highlight the importance of specialised training, considering the different needs of different sports and genders.

In recent times, there has been an increase in research focusing on the differences between limbs among soccer players [20,21]. In addition, research regarding CODD asymmetry and its effects on performance, especially in different types of jumps, remains limited. Some authors explored the relationship between CODD asymmetry and various bilateral jumps, finding that greater asymmetry could correlate with poorer jumping performance [20,21]. These results reinforce the notion that greater unilateral jumping ability and concentric strength could enhance COD performance, which is fundamental during the turning phases of specific tests such as the 505. On the other hand, Lockie et al. [22] and Dos’Santos et al. [23] did not identify significant correlations between CMJ asymmetries and physical fitness tests. Fort-Vanmeerhaeghe et al. [24] also found no significant associations between CMJ asymmetry and COD in mixed team sport participants. This aligns with the results presented by Bishop et al. [25] and Loturco et al. [26], who reported no association between vertical jump asymmetries and COD in elite female soccer players. These results suggest that the high level of both general and specific training among female soccer players may elucidate the influence of asymmetries on performance, mirroring the integral role of vertical jumps and COD actions in competitive soccer [25,27]. The paucity of studies on female soccer players further highlights the existing gap in sport science research, accentuating the demand for in-depth and gender-focussed analyses to further enhance training strategies and performance strategies in women’s team soccer.

In reviewing the existing literature, some studies have shown that male soccer players categorised by their CODD, particularly those who exhibit greater speed, tend to show lower performance in CODD metrics [28]. However, there is a notable lack of research on whether performance metrics related to acceleration, speed, and jumping can explain variations in CODD among female soccer players. Moreover, to date, no study has further explored the analysis of these variations in terms of percentage CODD (%CODD), a novel method for calculating the COD deficit proposed by Freitas et al. [14], and which aims to simplify its assessment, as there are currently two different calculations for CODD (time-based or speed-based), which can lead to confusion for physical trainers and coaches. This approach, which is based on the percentage difference between speed (linear sprint) and COD abilities rather than raw data, standardises this metric, thereby facilitating a more comprehensive understanding of COD ability in group comparisons [14]. Therefore, this study examined whether performance metrics based on speed and jumping could explain the variation in %CODD in young female soccer players.

## 2. Materials and Methods

### 2.1. Participants

Thirty-three highly trained adolescent female soccer players (age: 16 ± 0.95 years; height: 160.4 ± 5.22 cm; body mass: 55.7 ± 7.22 kg) agreed to participate in the study. A preliminary power analysis was performed to establish the necessary number of participants, utilizing G*Power software (version 3.1.9.3, based in Düsseldorf, Germany). Given the research structure, which examines variances within a single group, and taking into account an effect size of 0.5, a significance threshold (alpha) of 0.05, and a desired statistical power of 80%, it was determined that 27 subjects would be needed. However, the study included 33 individuals, thereby achieving a statistical power of 87%. These athletes had participated in soccer training at the club level for at least four years, performing three weekly technical and tactical sessions on the field of 90 min each and one match per week. In addition, they performed a weekly physical training session, lasting 90 min, focusing on speed, agility, quickness, injury prevention, and coordination to maintain their physical condition. Two teams from the same women’s football club participated in this study, competing in national (Third Spanish Women’s Division) and regional (First Regional League) competitions, respectively. Written informed consent was requested and obtained from the parents or guardians of all participants. The ethical guidelines of the Declaration of Helsinki (2013) were followed, and, in addition, approval was received from the local ethics committee (CP19/039, CEICA, Zaragoza, Spain).

### 2.2. Procedures

Physical fitness assessments were performed sequentially on the same day in the following order: unilateral horizontal and vertical jumps, linear sprint, and COD tests. It was recommended that no high-intensity activities be performed during the 48 h prior to the tests to minimize muscle fatigue. In addition, players were advised to avoid consuming caffeine or any other stimulant (i.e., energy drinks or dietary supplements) that could alter their natural performance. The importance of maintaining proper nutrition and optimal hydration 48 h before testing was also emphasised. All participants had previously performed these tests several times (i.e., at least five times), which ensured their familiarity with the procedures. To facilitate recovery and maintain consistency in performance, a 3 min rest interval was allowed between each test. Participants wore athletic shoes for the jumping tests and soccer boots for the speed (linear sprint) and COD testing. A warm-up protocol for the rise, activate, mobilize, and potentiate (RAMP) system was performed before the tests [29]. The “activation” phase consisted of dynamic movements and exercises specifically designed to activate the key muscle groups used in the tests. This was followed by the “mobilize” phase, which included dynamic stretching to improve flexibility and joint mobility. Finally, the “potentiation” phase was designed to prepare the neuromuscular system, incorporating specific exercises that imitated the movement patterns and intensity of the tests to be performed.

#### 2.2.1. Unilateral Countermovement Jump Test

The test was performed twice, with a 45 s rest interval between attempts, and the highest result was recorded. Participants were required to perform a jump on one leg, with the hands placed on the hips and the opposite leg flexed at a 90° angle at both the hip and knee for CMJ with a left and right leg (CMJL and CMJR, respectively) using the Optojump system (Microgate, Bolzano, Italy). A jump was considered invalid if the technique was incorrect, such as not keeping the hands on the hips or not landing on the same leg. Swinging the flexed leg in the air was permitted, and, upon landing, participants had to maintain balance on the leg they had jumped with for 3 s to ensure a valid attempt. The intraclass correlation coefficient (ICC) was 0.99, and the coefficient of variation (CV) was 3.6%.

#### 2.2.2. Unilateral Horizontal Jump Test

Performance (i.e., distance) in the left and right horizontal jumps (HJL and HJR) was measured using a tape measure over two attempts, with a 45 s rest between each jump. The longest jump was selected for analysis. Swinging the leg for momentum was allowed, and landing on one leg was required. Only jumps where the participant could maintain balance and hold the position at the end for 3 s were considered. The reliability of the test was high, with an ICC of 0.98 and CV of 1.6%.

#### 2.2.3. The 40 m Sprint Test

Running speed was assessed with a 40 m sprint, with partial times recorded at 10 and 30 m. Timing was performed using double-beam photoelectric cells (Witty, Microgate, Bolzano, Italy). The starting position required the leading foot to be positioned half a meter behind the initial timing gate, and a two-point staggered stance was adopted. The timing gates were set up with a separation of 1.5 m between them and at a height of 0.75 m. The placement of timing gates at varying distances made it possible to distinguish between acceleration capability (0 to 10 m) and maximum speed capability (30 to 40 m). Each participant completed the 40 m sprint twice, with a minimum of 3 min of passive recovery between attempts. The reliabilities were 0.96 and 1% for ICC and CV, respectively.

#### 2.2.4. The 180° COD Test

A 10 m shuttle sprint test was conducted. Participants sprinted from the start/finish line to the 5 m mark, touching it with either foot, and then made a 180° turn to sprint back. Participants had to ensure that the outside foot crossed the finish line to consider the trial successful. They started with the leading foot positioned half a meter back from the initial timing gate. Timing gates, located 0.75 m high and 1.5 m apart, were used to record the times (Witty, Microgate, Bolzano, Italy). Each player completed two trials, one with the left foot in front and the other with the right, in alternating order, with a two-minute rest between them. The fastest time for each foot was used for the analysis. The variables analysed included 180 COD with left (COD180L) and right (COD180R) legs. The ICC value was 0.80 and CV was 1%. The percentage-based COD deficit was calculated using the formula ((COD time − 10 m sprint time)/10 m sprint time) × 100)) [14].

### 2.3. Statistical Analyses

Statistical evaluations were conducted with the aid of SPSS (version 25, IBM, New York, NY, USA) and Microsoft Excel (version 2016, Microsoft Corp., Redmond, WA, USA). Data are presented as mean ± SD. Within-session reliability was assessed by CV and ICC using a spreadsheet specially designed for the calculations. The Shapiro–Wilk test was employed to evaluate normality, indicating that all variables followed a normal distribution, except for interlimb asymmetries. Multiple linear regression models were applied, using a backward stepwise elimination approach, with COD times and %CODD as dependent variables. The independent variables were anthropometry, unilateral vertical and horizontal jumps, 10 m and 30–40 m sprints, and limb differences. In the backward procedure, variables with *p*-values > 0.05 were removed from the model. Relationships between COD times and %CODD with the other variables were evaluated through Pearson’s product moment coefficient. Based on Hopkins et al. [30], the scale of correlation coefficients was classified as follows: trivial for *r* less than 0.01, small for *r* from 0.1 to less than 0.3, moderate for *r* from 0.3 to less than 0.5, large for *r* from 0.5 to less than 0.7, numerous for *r* from 0.7 to less than 0.9, nearly perfect for *r* from 0.9 to less than 1, and perfect when *r* equalled 1. Players were divided into good or bad %CODD using the median split technique. Differences between the two conditions (good vs. bad) were analysed using paired t-tests. Friedman’s analysis of variance was utilized to identify variations in asymmetry scores, establishing statistical significance at a *p*-value of less than 0.05. The size of the disparity between the two groups was determined by calculating Cohen’s d effect sizes using the equation (MeanCOD1 − MeanCOD2)/SDpooled, with COD1 and COD2 denoting the specific COD angles under consideration (for instance, COD45, COD90, COD135, and COD180). These were interpreted in line with Hopkins et al. [30] where <0.2 = trivial; >0.2–0.6 = small; >0.6–1.2 = moderate; >1.2–2.0 = large; >2.0–4.0 = very large; and >4.0 = near perfect.

## 3. Results

Descriptive physical performance and asymmetries data are reported in Table 1.

COD180L, COD180R, %CODDR, and %CODDL were significantly (*p* < 0.05) and largely (*r* = 0.63 to 0.82) related (Table 2). Furthermore, significant (*p* < 0.05) relationships were found between both COD180 and horizontal jumping with either right (*r* = −0.59 to −0.47) or left (*r* = −0.53 to −0.49) legs, 10 m (*r* = 0.53 to 0.70), and 30–40 m (*r* = 0.51 to 0.65) (Table 2).

The single best predictor of COD180 right and left was performance in the 10 m sprint, with and explained variables of 28% and 50%, respectively (*p* < 0.01). However, no regression model was predictive of %CODD on either limb (Table 3).

When the cohort was divided using a median split technique by the mean %CODD (Table 4), the subgroups exhibiting good %CODD were better in the HJ asymmetry, COD180R, 10 m, and %CODD for both right and left, with “moderate and large” ES’s ranging from 0.63 to 2.39.

## 4. Discussion

This study examined whether performance metrics based on speed and jumping could explain the variation in %CODD in young female soccer players. Regression analyses revealed the 10 m distance as a predictor of both legs in COD180; however, no predictors were identified for %CODD. These findings suggest that individual variance in COD180 right/left can be explained by the 10 m distance (acceleration phase). Significant correlations were found between COD180 right/left and %CODD right/left, 10 m, 30–40 m, and horizontal jump right/left, whereas %CODD right/left was only significantly related to COD times. When dividing the group by the %CODD performance, effect sizes indicated that the subgroup with good COD (indicating faster COD ability) exhibits a quicker COD180 change, has a lower %CODD, and shows a lower horizontal jumping asymmetry, indicating that the current female soccer players maintain greater mean speed during COD in both directions, as they could apply horizontal force symmetrically.

In soccer performance, the ability to accelerate in the fastest possible time is related to greater lower body power and, in turn, faster COD [31]. The current results indicate that a 10 m distance (acceleration) is predictive of better COD performance tests. Emmonds et al. [8] also reported that 10 m and 20 m times were good predictors of better COD performance in elite female soccer players. Acceleration has been described as a task that is more dependent on concentric propulsion [32], and maximal sprinting velocity seems to be more dependent on the stretch-shortening cycle (SSC) [33]. This implies that jumps in which countermovement is performed involve the SSC, which could lead to a lack of relationship between such jumps and shorter sprint distances (5 m to 20 m). This statement is reinforced by Young et al. [34], who identified a significant correlation between concentric strength during the squat jump (SJ) and 2.5 m sprint time, and by Baker and Nance [35], who reported similar significant results between concentric strength and 10 m sprint time, whereas this was not found for the 40 m sprint time. Another study in adolescent and collegiate female athletes provided moderate to numerous relationships between SJ and the 20 m sprint (*r* = −0.32 to −0.76) [35,36,37]. Regarding strength, Nimphius et al. [38] found moderate to numerous relationships (*r* = −0.50 to −0.75) between relative dynamic maximal strength (1 RM/Body Weight) and dominant leg COD performance in female soccer players. Therefore, it appears that concentric propulsion exercises such as plyometric split squats, jump squats, or box jumps focussing on the concentric phase of the movement may be useful in the design of training programmes aimed at improving acceleration and COD in female soccer players.

In the present study, significant relationships were found between COD180 and 10 m (*r* = 0.53 to 0.70) and 30–40 m (*r* = 0.51 to 0.65). In the same line, a recent study showed large correlations between 10 m speed and COD (Pro Agility test/5-10-5 *r* = 0.59; Zig-Zag test *r* = 0.55) [39]. In contrast, Lockie et al. [3] found heterogeneous results in collegiate women’s soccer players. In National Collegiate Athletic Association (NCAA) Division I players, the modified T-test was not significantly related to 10 m linear velocity (*r* = 0.18), and, on the other hand, the 505 test was positively correlated with 10 m sprint (*r* = 0.35). In NCAA Division II players, the modified *t*-test and 505 test showed a large relationship with 10 m sprinting (*r* = 0.66 and *r* = 0.55, respectively) [3]. No studies have been found in female soccer players analysing the relationship between COD and 30–40 m time. However, studies analysing the relationship between COD tests and 30 m time have been found. One study showed large correlations between 30 m speed and COD (Pro Agility test/5-10-5 *r* = 0.66; Zig-Zag test *r* = 0.55; 9-6-3-6-9 test *r* = 0.58) in elite female soccer players [39]. Another study analysed male soccer players and found a large correlation between linear speed (30 m) and the Zig-Zag test (*r* = 0.56–0.60) [40,41]. Therefore, further studies analysing the impact of maximal linear sprinting or long sprinting distances (40 m) and its relationships with the total distance covered, the distance prior to COD, and/or the number of CODs during a COD test should be considered in female soccer players.

In soccer, as in other sports, unilateral propulsion in the vertical and horizontal directions is a determinant in most actions. Previous studies have found high correlations between unilateral horizontal jumping and acceleration [42]. Kugler et al. [43] confirmed that horizontal forces are important for acceleration; however, forward propulsion requires maximal force in this direction and optimal horizontal force application. A recent systematic review and meta-analysis of highly trained athletes over 18 years of age indicated that horizontal jumping distance is positively associated with sprinting performance, showing that an athlete who jumps more may sprint faster [44]. According to this meta-analysis, this result is because both tasks involve several similarities, such as strength (horizontal force generation and power output) and movement characteristics (unilateral ground contacts and triple joint extension), among others [44]. Along the same lines, Robbins and Young [45] conducted an analysis of the possible relationship between sprinting ability (peak velocity or acceleration) and horizontal jumping in elite college soccer players, finding moderate correlation coefficients between long jumping distances and peak velocity performance (*r* = 0.35 to 0.47), and acceleration performance (*r* = 0.35 to 0.43). With reference to COD, it is likely that unilateral jumps (in horizontal, vertical, and lateral directions) resemble the action of changing direction itself [46]. In the current study, significant relationships were found between COD180 and HJR (*r* = −0.59 to −0.47) and HJL (*r* = −0.53 to −0.49). These findings concur with those reported by Lockie et al. [47], in which a significant relationship was observed between horizontal jumping and the 505 test in female university rugby players (*r* = −0.71). The same COD test was used to analyse these variables in male team sports players, also finding significant correlations between the HJR and 505 with both legs (left leg *r* = −0.30; right leg *r* = −0.48) and the HJL and 505 (left leg *r* = −0.37; right leg *r* = −0.53) [22]. Therefore, it is imperative to integrate unilateral horizontal and vertical jumping exercises into training programs to improve both acceleration and COD capabilities. Coaches and physical trainers are encouraged to focus on developing horizontal force production and optimization by taking advantage of drills that simulate real game situations to improve players’ ability to apply power effectively in multiple directions, such as lateral plyometric jumps, single-leg forward and backward jumps and lateral movements, ladder agility drills, or resisted sprints using bands or sleds to improve explosive power through forward propulsion.

A previous study recommended the use of CODD instead of normal COD to analyse this particular action and eliminate the first part of the linear sprint from the test (i.e., the time recorded in 10 m) [38]. However, two different calculations are currently proposed for the CODD (time-based or speed-based), which can lead to confusion for physical trainers and coaches. To standardise the measurement, Freitas et al. [14] proposed the use of % CODD, which is based on the percentage difference between the linear sprint and COD capacities. In the present research, this measurement was used, and it was observed that when dividing the sample into two groups (players with a better %CODD versus players with a worse %CODD), the subgroup with a good %CODD showed better results in the COD180R (ES = 0.98), the %CODD for both the right (ES = 1.80) and left legs (ES = 2.39), and the horizontal jumping asymmetry (ES = 0.73). Nevertheless, the group with the worst %CODD recorded a significantly lower time in the 10 m sprint (ES = 0.63). Fernandes et al. [28] analysed a sample of adolescent professional soccer players and divided them into two groups: those with better and those with lower CODD. In this study, players with better CODD showed significant correlations between CODS and CODD on both the left (*r* = 0.69) and right sides (*r* = 0.65). On the other hand, no significant associations were found between acceleration (10 m sprint) and CODD for either limb (left *r* = −0.34 and right *r* = −0.39). Other studies analysing male team athletes obtained similar results for both CODD and the COD 505 test (*r* = 0.74–0.81) and the 10 m sprint (*r* = −0.11 to 0.10) [38]. Similarly, the study by Lockie et al. [3] also identified that NCAA female soccer players with higher acceleration (10 m sprint) showed worse CODD results (NCAA Division I *r* = −0.88 and NCAA Division II *r* = −0.77). In relation to higher horizontal jumping asymmetry and lower COD performance, previous studies in male athletes have found associations between this higher HJ asymmetry and poorer COD ability (*r* = 0.60) [9]. Fernandes et al. [28] suggested that the possible reason why athletes who accelerate faster perform worse in the COD is that the greater the impulse, the greater the braking requirement, which depends on eccentric strength. Other studies have shown that COD performance is associated, to a greater extent, with eccentric strength than with isometric or concentric strength, mainly because of the importance of the braking phase mentioned above [48]. On this basis, training programmes that include exercises with eccentric loading (e.g., isoinertial training) to improve the specific technique of COD could be justified [11].

A possible limitation of this study is the fact that the sample was not classified according to playing positions due to the small number of players; however, this has been performed in previous research [27,49]. In addition, a larger number of players allows for more reliable conclusions to be drawn. Another limitation is the scarcity of literature related to %CODD, specifically in female soccer players. As the study focused on adolescent female soccer players, the data cannot be extrapolated to other populations such as men’s soccer, different age groups, or other team sports. In addition, consideration of the maturation stage can provide additional information. Despite these considerations, this study provides relevant data on the relationship between COD performance and other variables that are also key factors in soccer performance.

## 5. Conclusions

Acceleration in the first 10 m of a sprint is a strong predictor of COD180 performance, and individual differences in this test are largely explained by performance in these initial metres. No predictors were found for %CODD, but significant correlations exist between COD180 and %CODD performance, acceleration, linear velocity, and horizontal jump. In addition, %CODD correlates with COD180 times. Subgroups with better %CODD showed higher performance in COD ability, whereas those with lower %CODD showed poorer acceleration. In order to improve initial acceleration, deceleration, and COD, it would be advisable for coaches and trainers to include jumping exercises (in vertical and horizontal direction) with a higher eccentric load (e.g., isoinertial training) and agility exercises in training programmes. It would also be interesting for future studies to analyse whether performance metrics based on speed and jumping could explain the variation of %CODD in adult male football players and in male players of different ages.

## Figures and Tables

**Table 1:** Descriptive data for all physical performance tests.

Variable	Mean ± SD	Asymmetry (%)
CMJR (cm)	12.6 ± 2.19	10.2 ± 9.1
CMJL (cm)	12.4 ± 3.27	
HJR (cm)	129.0 ± 13.9	4.68 ± 7.08
HJL (cm)	126.8 ± 11.4	
COD180R (s)	2.97 ± 0.15	2.56 ± 1.67
COD180L (s)	2.97 ± 0.15	
10 m (s)	2.03 ± 0.07	
30–40 m (s)	1.47 ± 0.08	
%CODDR (%)	46.8 ± 6.44	7.85 ± 5.04
%CODDL (%)	46.3 ± 5.34	

CMJR and CMJL: unilateral countermovement jump with right and left legs; HJR and HJL: unilateral horizontal jump with right and left legs; 10 m: linear sprint of 10 m; COD180R and COD180L: 10 m shuttle sprint with one change of direction to right or left; %CODDR and %CODDL: percentage-based change of direction deficit to right or left.

**Table 2:** Pearson correlation coefficients (r) between COD, %CODD, and all performance scores for each test.

	COD180L	%CODDR	%CODDL	Body Mass	Height	CMJR	CMJL	Asy CMJ	HJR	HJL	Asy HJ	Asy COD	10 m	30–40 m	Asy %CODD
COD180R	0.82 (0.66; 0.91) *	0.71 (0.48; 0.85) *	0.63 (0.37; 0.80) *	0.15 (−0.20; 0.47)	−0.06 (−0.39; 0.29)	−0.19 (0.50; 0.16)	−0.21 (−0.51; 0.15)	0.00 (−0.34; 0.35)	−0.47 (−0.69; −0.15) *	−0.53 (−0.74; −0.22) *	−0.00 (−0.34; 0.34)	−0.14 (−0.46; 0.21)	0.53 (0.23; 0.74) *	0.51 (0.20; 0.72) *	−0.23 (−0.53; 0.12)
COD180L	___	0.35 (0.00; 0.62) *	0.72 (0.49; 0.85) *	0.33 (−0.02; 0.60)	0.04 (−0.31; 0.38)	−0.27 (−0.56; 0.08)	−0.17 (−0.49; 0.18)	−0.16 (−0.48; 0.18)	−0.59 (−0.77; −0.31) *	−0.49 (−0.72; −0.18) *	−0.17 (−0.48; 0.19)	−0.22 (−0.52; 0.13)	0.70 (0.48; 0.84) *	0.65 (0.39; 0.81) *	−0.27 (−0.56; 0.08)
%CODDR	___	___	0.71 (0.48; 0.85)	−0.08 (−0.41; 0.27)	−0.07 (−0.41; 0.28)	0.12 (−0.23; 0.45)	0.05 (−0.30; 0.39)	0.01 (−0.33; 0.36)	0.01 (−0.33; 0.36)	−0.12 (−0.44; 0.23)	0.34 (−0.00; 0.61)	0.03 (−0.31; 0.37)	−0.22 (−0.53; 0.13)	0.00 (−0.34; 0.34)	−0.09 (−0.42; 0.26)
%CODDL	___	___	___	0.16 (−0.20; 0.47)	0.05 (−0.29; 0.39)	0.03 (−0.32; 0.37)	0.09 (−0.25; 0.43)	−0.22 (−0.52; 0.13)	−0.19 (−0.50; 0.16)	−0.12 (−0.45; 0.23)	0.16 (−0.19; 0.48)	−0.09 (−0.42; 0.26)	0.01 (−0.33; 0.35)	0.23 (−0.12; 0.53)	−0.18 (−0.49; 0.17)

CMJR and CMJL: unilateral countermovement jump with right and left legs; Asy CMJ: between-limb CMJ asymmetry; HJR and HJL: unilateral horizontal jump with right and left legs; Asy HJ: between-limb HJ asymmetry; 10 m: linear sprint of 10 m; COD180R and COD180L: 10 m shuttle-sprint with one change of direction to right or left; Asy COD: between-limb COD asymmetry; %CODDR and %CODDL: the percentage-based change of direction deficit to right or left; Asy %CODD: between-limb %CODD asymmetry. * *p* < 0.05.

**Table 3:** Model linear regression analysis with measures of COD and %CODD.

		Variables	SC	Partial *r*	*p*	R^2^	*r*	Rating
COD180R	Model 1	Intercept 10 m	0.53	0.53	0.01	0.28	0.53	Moderate
COD180L	Model 1	Intercept 10 m	0.71	0.71	<0.01	0.50	0.71	Large

No variables in the %CODDR and %CODDL models. COD180R and COD180L: 10 m shuttle-sprint with one change of direction to the right or left; SC: standardised coefficient; %CODDR and %CODDL: the percentage-based change of direction deficit to the right or left.

**Table 4:** Analysis of all variables between the good and bad %CODD groups.

	Good %CODD (*n* = 16)	Bad %CODD (*n* = 17)	ES (CI95%)	*p*
Age (yr)	16.3 ± 0.93	15.7 ± 0.91	0.62 (−0.08; 1.32)	
Body mass (kg)	57.1 ± 6.67	54.3 ± 7.65	0.39 (−0.29; 1.08)	
Height (cm)	161.2 ± 4.93	159.6 ± 5.53	0.30 (−0.38; 0.98)	
CMJR (cm)	12.4 ± 2.32	12.6 ± 2.12	0.09 (−0.56; 0.77)	
CMJL (cm)	12.2 ± 3.68	12.6 ± 2.92	0.13 (−0.56; 0.81)	
Asy CMJ (%)	11.0 ± 11.1	9.50 ± 6.85	0.17 (−0.52; 0.85)	
HJR (cm)	128.0 ± 9.81	130.0 ± 17.1	0.14 (−0.54; 0.82)	
HJL (cm)	128.1 ± 12.7	125.7 ± 10.4	0.21 (−0.47; 0.89)	
Asy HJ (%)	2.14 ± 7.29	7.07 ± 6.14	0.73 (0.22; 1.43)	0.04 *
COD180R (s)	2.91 ± 0.12	3.04 ± 0.15	0.98 (0.25; 1.70)	0.008 *
COD180L (s)	2.92 ± 0.11	3.01 ± 0.18	0.53 (−0.17; 1.22)	
Asy COD (%)	2.72 ± 1.62	2.41 ± 1.75	0.18 (−0.50; 0.86)	
10 m (s)	2.05 ± 0.06	2.00 ± 0.08	0.63 (−0.08; 1.32)	
30–40 m (s)	1.47 ± 0.07	1.47 ± 0.10	0.01 (−0.67; 0.69)	
%CODDR (%)	41.7 ± 3.66	51.6 ± 4.50	2.39 (1.48; 3.28)	<0.001 *
%CODDL (%)	42.6 ± 2.94	49.8 ± 4.75	1.80 (0.97; 2.61)	<0.001 *
Asy %CODD (%)	8.82 ± 5.16	6.93 ± 4.91	0.37 (−0.32; 1.06)	

CMJR and CMJL: unilateral countermovement jump with right and left legs; Asy CMJ: between-limb CMJ asymmetry; HJR and HJL: unilateral horizontal jump with right and left legs; Asy HJ: between-limb HJ asymmetry; 10 m: linear sprint of 10 m; COD180R and COD180L: 10 m shuttle-sprint with one change of direction to right or left; Asy COD: between-limb COD asymmetry; %CODDR and %CODDL: the percentage-based change of direction deficit to right of left; Asy %CODD: between-limb %CODD asymmetry; CI: confidence intervals; ES: effect size. * indicates a significant difference between the good and bad %CODD groups (*p* < 0.05).

## Data Availability

The data from this research can be made available by the corresponding author following a justified request. Due to privacy concerns, the data are not accessible to the public.
